# Chronic intestinal pseudo-obstruction: associations with gut microbiota and genes expression of intestinal serotonergic pathway

**DOI:** 10.1186/s12866-024-03200-z

**Published:** 2024-02-02

**Authors:** Giulia Radocchia, Massimiliano Marazzato, Karim Ben Harbi, Elena Capuzzo, Fabrizio Pantanella, Roberto De Giorgio, Matteo Guarino, Anna Costanzini, Letizia Zenzeri, Pasquale Parisi, Alessandro Ferretti, Enrico Felici, Anna Teresa Palamara, Giovanni Di Nardo, Serena Schippa

**Affiliations:** 1 Department of Public Health and Infectious Diseases, Microbiology section, Sapienza University of Rome, Rome, Italy; 2 Department of Translational Medicine, University of Ferrara, Ferrara, Italy; 3 Department of Neurosciences, Mental Health and Sensory Organs (NESMOS), Faculty of Medicine and Psychology, Pediatric Unit, Sapienza University of Rome, Sant’Andrea University Hospital, Rome, Italy; 4 Paediatric Emergency Department, Santobono-Pausilipon Children’s Hospital, Naples, Italy; 5 Unit of Pediatrics, The Children Hospital, Azienda Ospedaliera SS Antonio e Biagio e Cesare Arrigo, Alessandria, Italy; 6 Department of Infectious Diseases, National Institute of Health, Rome, Italy

**Keywords:** Chronic intestinal pseudo-obstruction (CIPO), Gut microbiota, Serotonin pathway, Peristalsis, Dysbiosis

## Abstract

**Background:**

Pediatric chronic intestinal pseudo-obstruction (PIPO) is a rare disease characterized by symptoms and radiological signs suggestive of intestinal obstruction, in the absence of lumen-occluding lesions. It results from an extremely severe impairment of propulsive motility. The intestinal endocrine system (IES) jointly with the enteric nervous system (ENS) regulates secreto-motor functions via different hormones and bioactive messengers/neurotransmitters. The neurotransmitter 5-hydroxytryptamine (5-HT) (or serotonin) is linked to intestinal peristalsis and secretory reflexes. Gut microbiota and its interplay with ENS affect 5-HT synthesis, release, and the subsequent serotonin receptor activation. To date, the interplay between 5-HT and gut microbiota in PIPO remains largely unclear. This study aimed to assess correlations between mucosa associated microbiota (MAM), intestinal serotonin-related genes expression in PIPO. To this purpose, biopsies of the colon, ileum and duodenum have been collected from 7 PIPO patients, and 7 age-/sex-matched healthy controls. After DNA extraction, the MAM was assessed by next generation sequencing (NGS) of the V3-V4 region of the bacterial RNA 16 S, on an Illumina Miseq platform. The expression of genes implicated in serotoninergic pathway (*TPH1, SLC6A4, 5-HTR3* and *5-HTR4*) was established by qPCR, and correlations with MAM and clinical parameters of PIPO have been evaluated.

**Results:**

Our results revealed that PIPO patients exhibit a MAM with a different composition and with dysbiosis, i.e. with a lower biodiversity and fewer less connected species with a greater number of non-synergistic relationships, compared to controls. qPCR results revealed modifications in the expression of serotonin-related intestinal genes in PIPO patients, when compared to controls. Correlation analysis do not reveal any kind of connection.

**Conclusions:**

For the first time, we report in PIPO patients a specific MAM associated to underlying pathology and an altered intestinal serotonin pathway. A possible dysfunction of the serotonin pathway, possibly related to or triggered by an altered microbiota, may contribute to dysmotility in PIPO patients. The results of our pilot study provide the basis for new biomarkers and innovative therapies targeting the microbiota or serotonin pathways in PIPO patients.

## Introduction

CIPO is a rare gut disorder resulting from a severe impairment of propulsive motility, characterized by symptoms and radiological signs suggestive of intestinal obstruction, in the absence of lumen-occluding (mechanical) lesions. CIPO represents the most severe form of gastrointestinal (GI) dysmotility leading to potentially lethal complications (e.g. dehydration and electrolyte imbalance, ischemia/perforation and severe malnutrition) [1, 2]. Although GI dysmotility can affect the whole GI tract, the small intestine and colon are usually the most involved segments [2]. CIPO in the paediatric patients is defined by the acronym PIPO (Paediatric Intestinal Pseudo Obstruction), reflecting similarities with adult disease, but also distinctive features, as highlighted in a recent consensus paper [3]. In most cases of PIPO, intestinal and extra-intestinal (e.g. urinary and neurological) manifestations may occur in about 80% of patients in their first year of age [2]. In the remaining cases, symptoms appear in the first 20 years of life [4]. PIPO is generally idiopathic and may be related to various underlying abnormalities affecting the gut neuro-muscular component of the intestine, i.e., smooth muscle (myopathy), intrinsic (ENS) and extrinsic nervous system (neuropathy), ICC (mesenchymopathy) or with a mixed pattern, e.g. neuro-ICC-myopathy [5, 6]. Recurrence of sub-obstructive crisis can cause unnecessary (even life-threatening) surgical interventions complicated by possible adhesions that mix a mechanical component with existing dysmotility, thus increasing the morbidity and mortality associated with the disease [4, 7]. Apart from acute sub-obstuctive crisis, PIPO patients complain severe but unspecific digestive symptoms, leading to a long delay in diagnosis. Furthermore, the suboptimal efficacy of medical treatments, and the limited knowledge of the syndrome by physicians contribute to the poor quality of life and the high morbidity and mortality rate of these children [8]. Furthermore, PIPO patients complain of symptoms such as abdominal distension, dysphagia, abdominal pain, nausea, vomiting, bloating, constipation, diarrhoea and involuntary weight loss (usually very severe), which are known to be nonspecific as they overlap with other chronic intestinal disorders and lead to delays in diagnosis. Due to the severe alteration of gut motility, PIPO patients may be unable to maintain adequate oral feeding and growth, thus requiring enteral and/or parenteral nutritional support [2]. Since specific diagnostic tools are not available, the identification of PIPO/CIPO remains challenging for clinicians and may require several months or years before it is established. Therefore, both adult and paediatric patients require a multidisciplinary team of clinicians experienced in performing different tests (e.g. gastric emptying study, intestinal manometry, colonic transit study and electrogastrography), which could contribute with significant clinical information to the diagnostic process. It has been estimated that in the USA about 100 infants per year are affected by intestinal pseudoobstruction [9]. A Japanese survey observed a prevalence of 3.7 in one million children (1 in 270.000 children < 15 years of age) with no gender differences [10]. However, since symptoms are nonspecific and often physicians fail to establish an early diagnosis, the prevalence and incidence of PIPO/CIPO remain unclear [8]. To date, there are no definitive therapies for PIPO and treatments are mainly aimed at reducing symptoms severity [1]. Hydro-electrolyte repletion (in case of excessive dehydration), use of prokinetic agents (promoting intestinal motility) and antibiotics (to treat intestinal bacterial overgrowth) along with nutritional support are mandatory in a case-by-case evaluation. Parenteral feeding may be the primary intervention in the most severe PIPO patients refractory to any other therapeutic measures [1, 11–15]. Gastrointestinal (GI) motility is one of the most sophisticated physiological functions performed by the alimentary tract, beginning with esophageal peristalsis, gastric trituration and emptying of tiny particles into the duodenum, followed by absorption of nutrients throughout the length of the small intestine and, finally, expulsion of indigested residues by the large bowel. Clearly, this complex and highly integrated physiology requires a refined organization of various specialized key cell types [16]. More specifically: the smooth muscle, composed of two main layers throughout the GI tract (outer layer-longitudinal, inner layer-circular), provides the anatomical structure that mediates motility; the dense intrinsic (enteric) innervation, embedded in the wall of the GI tract, forms the enteric nervous system (ENS) integrated with the extrinsic parasympathetic and sympathetic pathways, responsible for neural input and activation; and the network of interstitial cells of Cajal (ICC) that performs various functions, including stimulation of the intestine. Also, the intestinal endocrine system (IES) is an important component for the regulation of intestinal motility; it acts jointly with the ENS and regulates the peristalsis by the secretion of different hormones and bioactive messengers/neurotransmitters, via the enteroendocrine cells (EECs) widespread throughout the intestinal mucosal lining [17]. The neurotransmitter 5-hydroxytryptamine (5-HT) (or serotonin) influences gut motility as well as bronchi, uterus and vessel contractility directly and via neural input [18]. 5-HT is a biogenic amine stored in a subset of the EECs, i.e. the enterochromaffin cells (ECs). In these cells, tryptophan is converted to 5-HT through the enzyme tryptophan hydroxylase 1 (TpH1) [19] and released upon mechanical, chemical, or neural stimulation. 5-HT exerts its various biological effects binding to seven subclasses of receptors. From a motility perspective, 5-HT3 and 5-HT4 receptors, respectively encoded by *HTR3A* and *HTR4* genes, contribute to regulate coordinated motor patterns and secretion [20]. The 5-HT reuptake is necessary to deactivate the molecule and is mediated by the specific serotonin transporter (SERT), coded by the gene *SLC6A4* and expressed by different serotonin cell targets including the mucosal epithelial cells [21]. Abnormalities in serotonin reuptake can alter enteric serotonergic signalling, leading to sensory, motor, and secretory dysfunctions in the gut. These abnormalities have been implicated in the pathophysiology of several disorders, such as irritable bowel syndrome (IBS) [21, 22]. Literature data indicate decreased intestinal motility in germ-free animals [23, 24] suggesting that gut microbiota, which includes bacteria, fungi, viruses, and bacteriophage, represents another important aspect that can influence human GI physiology, including gut immune system maturation [25], metabolism, and nutrition [26, 27]. In particular, the mucosa-adherent microbiota (MAM) plays an important role in maintaining intestinal homeostasis, due to its proximity to the mucosal immune system [28, 29]. In addition, several studies have revealed gut microbiota impact on the production of bioactive messengers, including 5-HT [30, 31]. The microbiota is able to interact with the 5-HT pathway [24, 32, 33] by stimulating its synthesis through shorty chain fatty acids (SCAFs) or bile acids [24, 34]. Therefore, the function and anatomy of the ENS can be influenced by the microbiota through the 5-HT release from ECs [35]. Any noxae perturbing the intestinal ecosystem integrity along with the ENS/extrinsic nerve pathways, the smooth muscle, and ICCs can result in the transition from a physiological to a pathophysiological condition. In this context, highlighting the interplay among ENS, 5-HT and gut microbiota in CIPO may open new opportunities to better understand how to recognize these difficult cases and manage them appropriately. Preliminary data by Gu and collaborators have reported significant symptoms relief in adult CIPO patients undergoing faecal microbiota transplantation (FMT), indicating a possible role of gut microbiota in the disease state [36]. In our previous review article [17], we reviewed the literature data on knowledge of the gut microbiota and the pathology of CIPO, focusing on the role of the ENS and the IES in intestinal motility, indicating the importance of further studies to better comprehend the causes of gut motility dysfunction in CIPO patients. The aim of the present study was to investigate associations between CIPO and: (a) MAM; (b) 5-HT-related genes expression, in PIPO patients. For this purpose, we characterized the MAM and evaluated the expression levels of the intestinal serotonin-related genes (*Tph1*, *SLC6A4*, *HTR3A* and *HTR4*) in patients with PIPO and in a group of subjects matched for sex and age, with a negative diagnosis of PIPO. Comparative and correlation analyses were performed to highlight the differences between the two study groups and the associations (positive or negative) between the collected data.

## Matherials and methods

### Study population

Seven clinically, radiologically, and monometrically characterized PIPO patients, aged 4–14 (median age 7.8) and seven healthy controls (HC) subjects, sex- and age-matched (median age 10.4) were enrolled at Children’s Hospital Cesare Arrigo (Alessandria, Italy). All the patients had a neuropathic PIPO at manometry, most frequently involving the small intestine and colon. Control subjects included only patients presenting with lower GI bleeding due to colonic inflammatory isolated polyps but without evidence of intestinal bowel disorders (IBDs) or other GI disorders. The exclusion criteria were acute and chronic inflammatory bowel diseases, metabolic disorders, diabetes mellitus, celiac disease, food allergies, anti-inflammatory drugs within 2 weeks prior to recruitment, probiotics/prebiotics/symbiotics in the past 30 days, antibiotics in the past 60 days, concomitant infectious diseases. All patients performed routine analysis (see below) and gastroduodenoscopic/colonoscopic examinations.

### Sample collection

PIPO patients and HC underwent routinary blood tests and, after informed consensus, upper/lower GI endoscopies under general anesthesia. To analyze the MAM and the expression of serotonin-related genes, biopsies from duodenum, terminal ileum and left colon were collected. From each location, two tissue specimens anatomically close of about 15 mg each, were collected. To preserve DNA and RNA’s structure, samples were stored at -80 °C in “All protect Tissue Reagent” (Qiagen, Hilden, Germany) until subsequent analysis.

### Total DNA extraction and NGS sequencing

In order to isolate only the mucosa-adherent bacteria, each biopsy had an initial lavage with 500 μL of saline solution 0.9% (PBS, Thermo Fisher Scientific, Waltham, Massachusetts, USA) added with dithioerythritol 0.016% (DTT, Sigma Aldrich, Germany), followed by three washes with PBS. The whole DNA was extracted from all collected samples using the DNeasy Blood & Tissue Mini kit (Qiagen, Hilden, Germany) according to the manufacturer’s instruction. To increase the Gram-positive bacterial yield, an additional step of 4 h incubation with lysozyme (20 mg/ml, Sigma-Aldrich, Milan, Italy) was added to DNA extraction protocol. The extracted DNA quality and concentration were checked using the Eppendorf BioPhotometer Spectrophotometer (Eppendorf, Germany). DNA integrity was visualized on a 1% agarose gel containing SYBR® Safe DNA GelStain (Invitrogen by Thermo Fisher Scientific, Waltham, Massachusetts, USA). All DNA samples obtained were normalized to a final concentration of 20 ng/μL and sent for the sequencing. The V3-V4 region of the bacterial 16 S rRNA gene was amplified and sequenced via Next Generation Sequencing (NGS), on an Illumina MiSeq 2 × 300 bp platform.

### Bioinformatic analysis

The bioinformatic and metagenomic analyses were carried out at the Department of Public Health and Infectious Diseases. After demultiplexing, reads were merged by using USEARCH v11 [37] with a minimum percentage identity of 90% between aligned sequences. Subsequently, the primer sequences were removed by using Cutadapt 2.1 [38] and the sequences were filtered in order to keep only those presenting a total expected error ≤ 0.8 and a size over a range of 400–460 bp. Sequences that passed the quality filter were imported in the software package Quantitative Insights into Microbial Ecology 2 (QIIME2) V18.6 [39] and passed to the Dada2 algorithm [40] for chimera-checking. Operational taxonomic units (OTUs) defined by a 97% of similarity were picked by clustering sequences with an open reference approach against the 97% clustered Greengenes rDNA reference database v13_8. In order to minimize artifact, OTUs found in only one sample and/or presenting < 10 sequences across the whole population were filtered out. The α-diversity (within sample diversity) was measured by computing the Shannon index and the number of observed OTUs while the β-diversity (between samples diversity) was assess using the Bray-Curtis dissimilarity measure. The taxonomy assignment of OTUs was carried out by using a Naive Bayes classifier trained on a custom 97% clustered version of the Greengenes rDNA v13_8 reference database in which the sequences have been trimmed to only include the V3-V4 regions. OTUs not identified at species level and/or identified with a confidence < 0.75, using the reference database, were assigned to the deepest taxonomic level on the base of BLAST results obtained by querying sequences against available published data and taking only those results in agreement to the taxonomy assigned by the Naive Bayes classifier approach if a confidence > 0.75 were determined. For each colon district, a correlation network was computed separately. Briefly, a first filtering step was performed by removing OTUs present with a mean relative abundance of < 0.01% across all population. Subsequently, for each separate group, OTUs with a count of zero were filtered out and the remaining unique entries were tested for their correlations by using CoNet v1.1.1 [41] available as an application in Cytoscape [42]. The following combination of methods, Pearson correlation, Spearman rank sum correlation, Bray-Curtis dissimilarity, and Kullback-Leibler divergence, was used with a cutoff threshold of 0.5 for both positive and negative values (− 0.5 ≥ correlation ≥ 0.5) for all considered metrics in order to overcome weakness presented by the use of a single metric respect to compositionality, matching zeros and sample size. Only correlations supported by at least two different correlation metrics were retained. The Statistical significance of each pair was tested using 500 row shuffle randomizations followed by 100 bootstraps. The *p*-values relative to multi-edges connecting the same node pair were merged using the Fisher’s method and the merged *p*-values were corrected for multiple comparisons. In each randomization round a sample-wise normalization step was performed for each item pair in order to account for compositionality bias. Topological parameters were calculated for each computed network by using the Network Analyzer plugin included in Cytoscape.

### Total RNA extraction and retro-transcription

Total RNA was extracted from the second biopsy collected of each sample using the RNeasy Mini Kit (QIAGEN, Hilden, Germany). RNA concentration and purity were detected using the Eppendorf BioPhotometer Spectrophotometer. The mRNA was then converted in cDNA by the high efficiency retro-transcription kit “Quanti Nova Reverse Transcription Kit” (QIAGEN, Hilden, Germany) to investigate the expression of genes related to the serotoninergic pathway. A control reaction without reverse transcriptase (no-RT control) was performed to preclude any DNA contamination through the subsequent PCR analysis. cDNA was used as template for the quantitative Real Time PCR (qRT-PCR) analysis.

### Expression analysis of serotonin related genes

To assess the expression of serotonin related genes, the qRT-PCR was performed. The genes investigated were Tryptophan hydroxylase 1 (*TpH1*), responsible for 5-HT synthesis in the gut district; serotonin transporter (*SLC6A4*), which regulates the recaption of 5-HT at luminal level; *HTR3A* and *HTR4*, coding for the intestinal receptors of serotonin. qRT-PCR reactions were conducted in 96-well plates using the Applied Biosystems’s StepOnePlus Real Time PCR System (Applied Biosystem, CA, USA). Specific primers for the selected serotonin-related genes were provided by Qiagen (QuantiNova LNA PCR assay) and the Quantitect RT-PCR kit (QIAGEN, Hilden, Germany), with a Master Mix containing SYBR Green as fluorescent dye, was used for the qPCR reaction. Parameters set for the qRT-PCR were 95 °C for 2 min, then 40 cycles at 95 °C for 10 s and finally at 60 °C for 30 s. Each reaction was repeated in triplicate and the absence of contaminants was assessed substituting cDNA with Ultrapure water (negative control). The relative expression of each gene was determined via the ∆∆CT method [43]. qRT-PCR results were graphed using the statistical program “GraphPad Prism 8.0.1”.

### Statistical analysis

The X^2^ test was used to determinate statistically significant differences for the discrete variables. The Mann-Whitney test was used for pairwise comparisons while the Kruskal-Wallis test followed by the Dunn’s post hoc test was performed for multiple comparisons. The bacterial community composition among samples was compared by the Principal Coordinate Analysis (PCoA). The statistically significance of partitions between groups was evaluated with the Permutational Analysis of Variance (PERMANOVA), while correlations between the relative abundances of taxa (at species levels), the considered clinical variables and the expression of 5-HT related genes have been determined by the Spearman’s rank correlation computed with the Vegan package in R. In order to decrease the false discovery rate, the computed *p*-values have been corrected by the Benjamini-Hochberg procedure to consider for multiple comparisons. In each case, the significance level was set for *p* value < 0.05.

## Results

### Clinical parameters

Evaluation of clinical parameters showed that only the ferritin value was significantly lower in PIPO patients vs. HC (*p* < 0.01), suggesting a homogeneous population, in relations to the clinical parameters considered. Median, interquartile range (IQR) and significance of all clinical parameters and demographic variables (i.e. sex, age and weight) are summarized in Table 1.

**Table 1:** Demographic and clinical variables relative to the studied population of subjects. *significance (*p* < 0.01)

	PIPO patients (No. 7)	Control patients (No. 7)	*p*-value
Sex male n (%)	5 (71%)	2 (29%)	0.1263
Age (years) Median (IQR)	8.0 (14–5)	10.0 (14–5)	0.3057
Weight (Kg) Median (IQR)	25 (35–15)	42 (69–12.5)	0.0728
PCR (mg/dl) Median (IQR)	0.115 (0.53–0.01)	0.05 (0.28–0.04)	0.7668
Hb (g/dl) Median (IQR)	12.75 (14 − 12.225)	12.5 (14.1 − 12.2)	0.8497
Ht (%) Median (IQR)	39.5 (42–36)	37 (41 − 36)	0.3153
Ferritin (ng/ml) Median (IQR)	32.5 (48–12)	73 (90–51)	0.0042*
Calprotectin (mg/kg) Median (IQR)	64 (72–51)	76 (82–34)	0.7928
Total proteins (g/dl) Median (IQR)	6.5 (6.5–6.2)	6.7 (6.9–6.1)	0.5016
Albumin (g/dl) Median (IQR)	4.3 (4.6–4)	4.6 (4.7–4.3)	0.1104

### Mucosa-adherent microbiota characterization

**Fig. 1:**
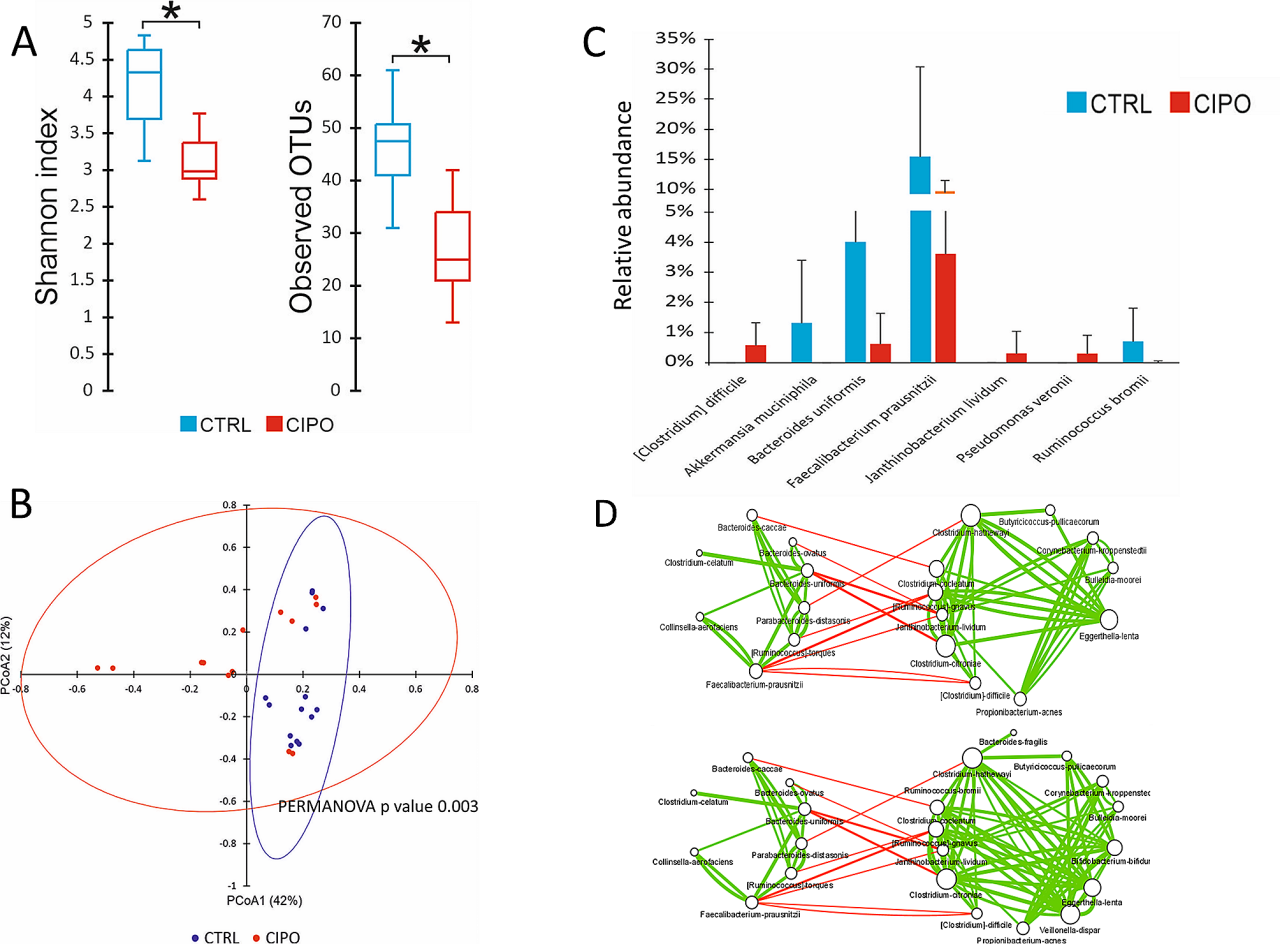
Changes in microbiota composition and microbial relationships at the colon level. (**A**) Color-coded box and whisker plots showing the distribution of the alfa-diversity estimators among PIPO and Non PIPO patients. * Statistical significance at alpha level 0.05. (**B**) PCoA analysis performed for β diversity based on the Bray–Curtis measure of dissimilarity. For each principal coordinate, the percentage of variance explained is reported between parentheses. Ellipses represents 95% confidence intervals. (**C**) Color-coded bar plot showing differential abundance analysis at genus and species levels performed by Mann–Whitney U tests. Only taxa whose abundances were significantly different between groups at alpha level 0.05 were reported. Error bars: SD. (**D**) Co-occurrence networks in PIPO patients (up) and control patients (down). Nodes: species. The size of nodes is proportional to number of connections with other nodes in the networks. The thickness of edges is proportional to the strength of correlations between species. Red edges: negative correlations. Green edges: positive correlations

Starting from a total of 1.254.322 raw sequences, the initial filtering steps led to the determination of 304.543 high-quality sequences grouped in 159 OTUs that have been assigned to 44 bacterial genera and 29 different species. Statistical differences were detected only at colon level. Evaluation of α-diversity, by the Shannon index and the number of observed OTUs, showed a significant lower biodiversity in PIPO patients vs. HC (Fig. 1.A). The β-diversity analysis showed statistically significant partition between PIPO *vs.* HC (Fig. 1.B). Differences in the relative abundance of taxa were determined by considering taxonomic groups with an average relative abundance ≥ 0.5% in at least one of the two analysed groups. Bacterial species commonly considered beneficial and generally associated with an health status, i.e. *Akkermansia muciniphila*, *Ruminococcus bromii*, *Bacteroides uniformis* and *Faecalibacterium prausnitzii*, were significantly more abundant in HC than in PIPO patients. On the other hand, *Clostridium difficile (*often considered potential pathogen), *Janthinobacterium lividum* and *Pseudomonas veronii*, were significantly more abundant in PIPO group compared to HC, where they were totally absent (Fig. 1C).

### Network analysis

The interactions between taxa in the two groups were investigated by network analysis. This analysis was performed only in the colon district, where we found significant differences in MAM α- and β-diversity. The microbial networks obtained for PIPO patients and HC are represented separately in Fig. 1D. In both patient and HC networks the presence of two microbial modules has been highlighted, denoting associations of microorganisms that are somehow related in the functions they perform in the ecosystem. The taxa inside the modules correlated with positive interactions, reflecting synergic interactions, while modules are related to each other with antagonistic interactions, showing competitions between microbes. The topological properties of co-occurrence networks (Table 2) indicate fewer species and fewer connections between taxa associated with PIPO patients, compared with the HC network (Fig. 1D), with negative interactions (antagonistic interactions) more abundant in PIPO than in HC (Fig. 1.D). A decrease in mutualistic/beneficial relationships among bacteria in PIPO group in respect to control, it is a sign of microbial dysbiosis. We evidenced the same keystone species, so named because of their numerous interconnections with other microbes, in PIPO and control groups networks. However, their relative abundance was significantly different in the two groups studied; in particular, *C. difficile* and *J. lividum* were significantly more abundant in PIPO patients, while *B. uniformis* and *F. prausnitzii* were significantly more abundant in the HC.

**Table 2:** Topological properties of co-occurrence networks obtained for PIPO patients and the control subjects

	Control subjects	PIPO patients
Number of nodes	23	18
Number of edges	87	50
Edges to nodes ratio	3.78	2.7
Positive to negative edges ratio	7.7	4.1
Network density	0.32	0.25

### Serotonin pathways genetic expression

**Fig. 2:**
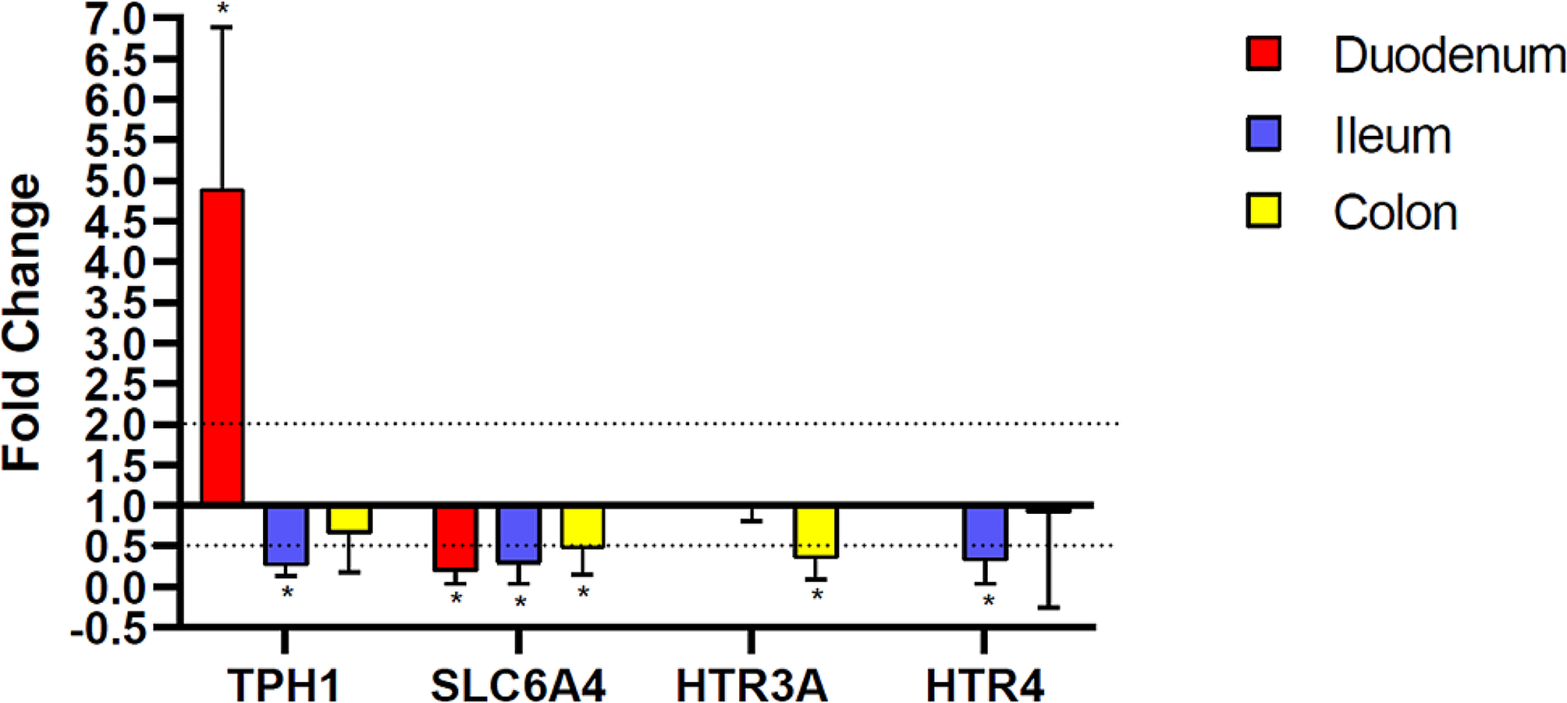
Fold change of serotonin-related genes (*TpH1*, *SLC6A4*, *HTR3A* and *HTR4*) in PIPO patients compared to controls in the different intestinal districts. The basal expression of 5-HT related genes in controls has a fold change of 1. The expression in PIPO patients is normalized with the basal expression of controls and the average fold changes are shown in the graph. All the over-expressions are represented up the basal line and all the under-expressions are represented down the basal line. The up- and down- regulation are considered statistically considerable for fold change values over 2 and under 0.5, respectively. Error bars: SD, *n* = 7

The qRT-PCR revealed a decrease in the expression of serotonin-related genes in PIPO patients *vs*. HC in almost all the anatomical location analysed. The expression profiling of up- and down-regulated genes is represented in Fig. 2, while the specific averages of fold changes are reported in Table 3. Our results highlighted, in PIPO group, an increased expression of *TpH1* gene in the duodenum, while the *SLC6A4* expression was decreased, and no expression detection of the genes *HTR3A* and *HTR4 *was revealed. In PIPO ileum district all genes assayed were significant decreased in their relative expression compared to HC, except for *HTR3A*. Regarding the colon, the relative expression of all genes analysed was decreased in PIPO patients *vs.* HC, reaching the statistical significance only for *SLC6A4* and *HTR3* genes. The expression analysis revealed a general alteration in the serotonin pathway in PIPO patients *vs.* HC, also depending on the intestinal district, where different levels of genes expression were shown. Correlation analysis between the expression of serotonin-related genes and MAM do not showed connections among data.

**Table 3:** Average fold changes of selected serotonin-related genes in PIPO patients compared to controls in different intestinal districts. N.d.: non-detected

Gene	Duodenum	Ileum	Colon
*TpH1*	4,351843918	0,274784465	0,667919067
*SLC6A4*	0,203447837	0,299733093	0,476693965
*HT3RA*	n.d.	0,981906522	0,361638167
*HT4R*	n.d.	0,327973728	0,92170625

## Discussion

The present study aimed to evaluate whether a specific MAM could be linked to PIPO and whether dysfunctions in the serotonin pathway occurred. In PIPO patients, NGS analysis confirmed the presence of a specific MAM in colon district, with significant differences in α-diversity and β-diversity when compared to HC group. In particular, in the colon of PIPO patients, the relative abundance of taxa revealed a decrease of beneficial species such as *(A) muciniphila, R. bromii, (B) uniformis* and *F. prausnitzii*, while a higher presence of the potentially pathogenic species, e.g. *(C) difficile*, was observed. A reduction of *A. muciniphila* has been observed in IBD and metabolic disorders, suggesting its potential anti-inflammatory role in pathological conditions, from inflammation (IBD) to extra-digestive metabolic disorders [44]. Indeed, *A. muciniphila* is inversely associated with obesity, diabetes, cardiometabolic disease, and inflammation [45]. Similar considerations can be drawn for *F. prausnitzii*, defined as anti-inflammatory bacteria [46]. In addition, both *(A) muciniphila* and *F. prausnitzii* are known to produce butyrate and other SCFAs through fermentation of dietary fibers [47]. *(B) uniformis* reduces cholesterol and triglyceride concentrations, diminishes blood glucose levels, as well as insulin and leptin [48]. Network analysis investigated the connections between microbes. In the colonic MAM ecosystem, network analysis highlighted two modules of connections between microorganisms, both in PIPO and in HC group. Taxa within the modules were positively correlated with each other, while antagonistic connections were evidenced between the two modules (Fig. 1D). Although the key species that connect the two modules are the same in PIPO and HC (*C. difficile, F. prausnitzii*, several *Bacteriotedes* species, *B. uniformis* and *J. lividum*), they significantly differ in relative abundance in the two groups, therefore network analysis results corroborate NGS results. In PIPO patients, according to NGS results, the relative abundances of the potentially pathogenic species *(C) difficile* increased, while the relative abundances of *F. prausnitzii* and several *Bacteriotedes* species decreased (Fig. 1): those species resulted to be negatively related by network analysis. Differently, in the control group, by NGS analysis, we observed an increase of *B. uniformis* and *F. prausnitzii* species and a decrease of *C. difficile* and *J. lividum*, all species resulted negatively connected in network analysis. In addition, the PIPO network showed less connections (especially synergistic ones) and fewer species than the controls. Loss of mutualistic/synergistic connections is indicative of dysbiosis, as well as loss of biodiversity and the increase in potentially pathogenetic species. In the present study, we reported for the first time in PIPO patients a characteristic MAM in the colon with signs of dysbiosis. Analysis of the expression of serotonin-related genes highlighted an alteration of 5-HT pathway in PIPO patients *vs. *controls. In almost all segments considered, we observed a general and lower expression of the investigated genes. The only exception occurred in the duodenum where the *TpH1* gene was significantly over-expressed with concomitant under-expression of *SLC6A4*. This finding supports the 5-HT deficiency in the distal, medial, and proximal colon, but not in the small intestine, observed in germ-free mice, which suggests a specific role of microbiota in the regulation of 5-HT in the colon [31]. In this latter study, a reduced expression of the *TpH1* gene was associated with elevated colonic expression of the 5-HT transporter *SLC6A4* in germ-free mice [31]. The authors hypothesized a compensatory effect between the expression of these two genes, which was confirmed by chemical inhibition of *TpH1* expression on germ-free mice that modulated the expression of *SLC6A4*. In other words, the expression level of these genes (*TpH1* and *SLC6A4*) seemed to be negatively correlated: a decrease in *TpH1* expression was followed by an increase in *SLC6A4* expression, and vice versa. In our results, we observed this compensatory effect only in the duodenal district. The expression of 5-HT3 and 5-HT4 receptor genes (which are essential for proper peristalsis function) was generally reduced in all the districts analysed, with a significant decrease in the ileum for the gene *5-HT4* and in the colon for the gene *5-HT3*. Our results led us to hypothesize that PIPO pathophysiology is associated with alteration of the serotonin pathway, as it was demonstrated for other GI disorders [49, 50]. Although no correlations have been evidenced among MAM and serotonin related genes, we can’t exclude that such result could be due to the limited sample size analysed not permitting to reach statistical significance. Finally, among the clinical parameters analysis we highlighted significantly difference only for the ferritin amount, which was lower in PIPO patients than in HC, indicating the level of serum ferritin as a possible marker of the disease. Specifically, a low ferritin level indicates poor iron reserve in patients and could be a consequence of impaired iron absorption or blood loss, mostly subtle but chronic: this condition is often present in subjects with GI disorders. The link between ferritin and microbiota has been demonstrated in several studies, indicating that alteration of microbiota could decrease ferritin production [51]. Moreover, Deschemin and colleagues showed that, in germ-free mice, ferritin level was lower than in healthy mice [52], hypothesizing that iron level is affected by bacterial metabolism and vice versa.

## Conclusion

In our work, we demonstrated, for the first time that PIPO patients exhibit a specific colonic MAM profile that reflects a dysbiotic environment. The study also showed a general decrease in the expression of serotonin-related genes, which were also found to be differently expressed throughout the intestine. This result indicates that an altered serotonin availability may contribute to the gut dysfunction in PIPO. Although further studies with a larger sample size will be necessary to confirm the results of this research, our work represents a first step in delineating the pathophysiology of intestinal dysmotility in PIPO. Finally, our data may help to identify new disease markers and therapeutic targets useful for new pharmacological treatments, also focused on reconstituting the gut microbiota in PIPO.

## Data Availability

The datasets used and/or analysed during the current study are available from the corresponding author on reasonable request.
